# A Sustainable Approach to the Comparative Evaluation of Mechanical Surface Performance in Pigmented Paint and Transparent Varnish Systems Applied on Wood Panels

**DOI:** 10.1021/acsomega.6c04225

**Published:** 2026-07-09

**Authors:** Serdar Kaçamer

**Affiliations:** Department of Design, Bolu Abant Izzet Baysal University, Bolu 14030, Turkey

## Abstract

Selecting appropriate coating chemistry is essential for extending the service life of wood products and improving resource efficiency in sustainable manufacturing. This study comparatively evaluated the mechanical surface performance of transparent varnish and white pigmented paint systems applied to Eastern beech (*Fagus orientalis* L.) wood panels. Four representative coating chemistries—acrylic, polyurethane, water-based, and cellulosic—were investigated for both transparent and pigmented formulations. Mechanical performance and damage evolution were characterized using König pendulum hardness testing, scratch resistance testing, and scanning electron microscopy (SEM). Statistical analyses, including analysis of variance (ANOVA), Duncan’s Multiple Range Test (DMRT), and correlation analysis, were performed to assess the significance of coating chemistry and coating architecture on surface performance. The results demonstrated that acrylic and polyurethane systems exhibited superior hardness and scratch resistance compared with water-based and cellulosic coatings. Furthermore, transparent varnish consistently outperformed their pigmented counterparts within the same resin chemistry, indicating that coating architecture significantly influences mechanical durability. SEM observations revealed that scratch-induced damage mechanisms, including crack initiation, localized deformation, and coating fracture, were strongly dependent on coating composition and microstructural characteristics. The findings indicate that acrylic and polyurethane transparent varnish systems provide the highest mechanical durability and surface protection performance, supporting extended service life and improved resource efficiency in wood finishing applications.

## Graphical abstract

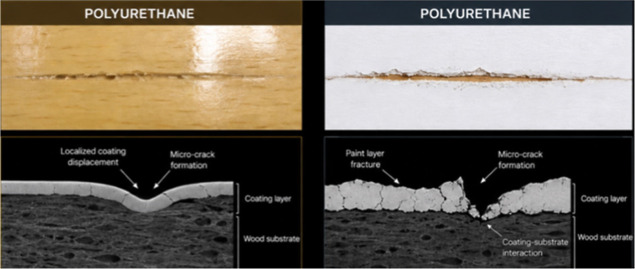

## Introduction

The transition toward a circular bioeconomy has intensified interest in renewable and sustainable materials capable of reducing dependence on fossil-based resources. Among such materials, wood occupies a strategic position due to its carbon sequestration potential, favorable strength-to-weight ratio, recyclability, and widespread industrial availability. Eastern beech wood (*Fagus orientalis* L.) is particularly valued in furniture, interior applications, engineered wood products, and decorative panels because of its homogeneous texture, dimensional stability, and favorable machining characteristics. Nevertheless, despite these advantages, wood remains susceptible to surface degradation arising from mechanical wear, scratching, abrasion, moisture fluctuations, and environmental exposure, which may significantly reduce the product service life. Consequently, extending the durability of wood-based products through effective surface protection strategies represents an important component of sustainable material management and resource-efficient manufacturing.^1–10^

To enhance the durability and aesthetic quality of wood products, protective coating systems are routinely applied to the surface. Wood coatings serve as functional barriers that reduce mechanical damage, moisture uptake, staining, and environmental deterioration while simultaneously improving appearance and surface quality. Among commercially available technologies, acrylic, polyurethane, water-based, and cellulosic coating systems are extensively employed in furniture and interior applications. These coating chemistries exhibit distinct film-forming mechanisms, cross-linking densities, and mechanical behaviors, resulting in substantial differences in hardness, scratch resistance, flexibility, and long-term durability. Consequently, selecting an appropriate coating chemistry is a critical factor in maximizing the service life and sustainability of coated wood products.^10–15^

Wood finishing systems are commonly classified into transparent varnishes and pigmented paints. While transparent varnishes primarily aim to preserve the natural appearance of wood, pigmented coatings provide enhanced color uniformity and conceal substrate-related visual imperfections. Despite often being formulated from similar resin chemistries, these two coating architectures differ fundamentally in their internal structure. The incorporation of pigment particles and extenders into paint formulations introduces additional interfaces within the polymer matrix that may alter stress distribution, crack initiation behavior, and scratch resistance mechanisms. Although numerous studies have investigated the mechanical performance of individual coating chemistries, direct comparisons between pigmented and transparent systems based on identical resin technologies remain limited. Consequently, the influence of coating architecture on the mechanical durability of wood finishing systems has not yet been fully clarified.^10,16–18^

Mechanical durability of coating films is commonly assessed through hardness and scratch resistance measurements. Pendulum hardness testing evaluates the damping behavior of an oscillating pendulum resting on the coating surface and provides an indirect measure of viscoelastic surface stiffness and cohesive integrity. In contrast, scratch resistance testing directly examines the ability of a coating film to withstand localized mechanical loading generated by a stylus moving across the surface under controlled conditions. Because scratch-induced failure involves complex interactions among hardness, elasticity, fracture resistance, and coating–substrate adhesion, combining pendulum hardness and scratch resistance measurements enables a more comprehensive assessment of coating performance. Furthermore, recent advances in surface characterization have highlighted the value of SEM observations for investigating deformation behavior, microcrack initiation, coating fracture, and coating–substrate interactions associated with scratch-induced damage.^17,19–22^

Among the various methods available for evaluating coating performance, König pendulum hardness and scratch resistance testing are widely recognized in both industrial practice and international standards as key indicators of surface mechanical durability. The König pendulum method, standardized under ASTM D4366, is extensively employed in furniture, flooring, architectural wood products, and industrial coatings to assess coating hardness through damping behavior without causing visible surface damage. Similarly, scratch resistance testing, standardized under ASTM D7027, is routinely used to evaluate a coating’s ability to withstand localized mechanical stresses that may occur during handling, service, transportation, and daily use. Because these methods characterize different but complementary aspects of coating performance, they are frequently incorporated into industrial quality-control protocols and product qualification procedures for wood finishing and protective coating systems. Therefore, the combined use of pendulum hardness and scratch resistance testing provides a practical and industry-relevant framework for assessing the long-term mechanical durability of transparent varnish and pigmented coating systems.^19,20^

Despite extensive research on wood coating technologies, a comprehensive comparison of transparent varnish systems and pigmented paint systems based on identical resin chemistries remains largely absent from the literature. Moreover, limited information is available regarding the relationship between coating architecture, scratch-induced damage mechanisms, and overall mechanical surface durability. Therefore, the objective of this study was to comparatively evaluate the mechanical surface performance of acrylic, polyurethane, water-based, and cellulosic transparent varnishes and pigmented wood coatings applied to Eastern beech (*F. orientalis* L.) wood panels. Pendulum hardness (ASTM D4366) testing and scratch resistance (ASTM D7027-20) measurements were employed to quantify surface durability, while SEM analysis was used to investigate scratch-induced damage mechanisms, including groove morphology, crack initiation, coating fracture, delamination behavior, and coating–substrate interaction. Statistical evaluation was performed using analysis of variance (ANOVA) and Duncan’s multiple range test (DMRT) to determine the significance of coating chemistry and coating architecture on mechanical performance. The findings are expected to provide scientifically grounded guidance for selecting coating systems capable of extending product service life, improving resource efficiency, and supporting sustainable wood utilization strategies.

## Material and Method

### Wood Specimens and Conditioning

To minimize substrate-related variability during coating performance evaluation, Eastern beech (*F. orientalis* L.) was selected as the wood substrate because of its uniform texture, moderate density, and widespread use in furniture and decorative panel production. Specimens were prepared from defect-free boards exhibiting straight grain orientation and free from knots, reaction wood, checks, or other structural imperfections, following the recommendations of ASTM D7787-13 (2022).^23^ The prepared panels measured 100 mm × 100 mm × 10 mm (*L* × *T* × *R*). Before coating and testing procedures, all specimens were conditioned in a climate-controlled chamber maintained at 20 ± 2 °C and 65 ± 3% relative humidity until moisture equilibrium was established. The conditioning process followed ISO 554 (1976), yielding a final moisture content of approximately 12%.^24^

Coating penetration into the cellular structure of wood may influence coating anchorage and stress transfer under mechanical loading, and a standardized sanding and surface preparation protocol was applied to all specimens before finishing. Prior to the coating application, all specimen surfaces were mechanically prepared to minimize variability associated with roughness and coating penetration. The wood panels were sanded using P150 grit abrasive paper along the grain direction and subsequently cleaned with compressed air to remove the sanding residues and loose particles. This surface preparation procedure was implemented to promote uniform coating penetration, improve coating adhesion, and reduce the influence of substrate-related variability on the measured mechanical performance. Standardized surface preparation is particularly important for diffuse-porous hardwood species such as eastern beech (*F. orientalis* L.), where surface texture and porosity can affect coating film formation and subsequent hardness and scratch resistance behavior.

### Transparent Varnish and Pigmented Paint Application

In this study, four transparent silk-matte transparent varnish systems widely applied in the furniture sector were investigated, including polyurethane (UN215), acrylic (UN315), water-based (UN531), and cellulosic (UN615), all supplied by UNLU Co. (Turkey). In addition, four industrial-grade silky matte white pigmented paint systems from the same manufacturer were used for panel finishing: a two-component polyurethane system (211-1002), a two-component acrylic system (311-1003), a two-component waterborne system (531-1002), and a single-component cellulosic system (611-1001), serving as primer/topcoat formulations. All painting and varnishing procedures were conducted in accordance with ASTM D3023-98 (2017) standard conditions.^25–27^

These eight coating systems were selected to represent the most used wood-finishing chemistries in both industrial and academic applications. Polyurethane and acrylic systems were included as high- and medium-performance cross-linked coatings widely used in furniture and interior applications. Water-based systems were selected due to their increasing industrial relevance as environmentally friendly alternatives with reduced volatile organic compound (VOC) content. Cellulose-based coatings were included as a conventional and cost-effective reference system representing traditional wood-finishing technologies. Together, these materials provide a representative performance spectrum ranging from conventional to advanced coating systems.

Prior to varnish and paint application, wood panel surfaces underwent preparation by sanding with P150 grit sandpaper and cleaning via compressed air to eliminate dust particles. Coating deposition was carried out utilizing an HVLP (high-volume low-pressure) spray system with a 1.6 mm nozzle diameter under an operating pressure of 2.5 bar and a tip opening of 1.6 bar. The varnish and paint were applied at a coverage rate of 125 g/m^2^ per layer in compliance with standard protocols. The pneumatic gun was moved uniformly at a constant velocity, parallel and perpendicular to the substrate, maintaining a consistent discharge distance of 20 cm.

Each primer coat paint and varnish sample was treated with a dual-layer primer filler, accompanied by intercoat sanding using P240 grit abrasives. Subsequently, two layers of silk matte varnish and white pigmented paint were applied as topcoats, incorporating a 20 min flash-off interval between the coats. Full cross-linking and film formation were achieved by curing the coated specimens under ambient conditions for 7 days. For each experimental group, ten replicates were fabricated to ensure reproducibility and facilitate statistical analysis (Figure 1).

**Figure 1:**
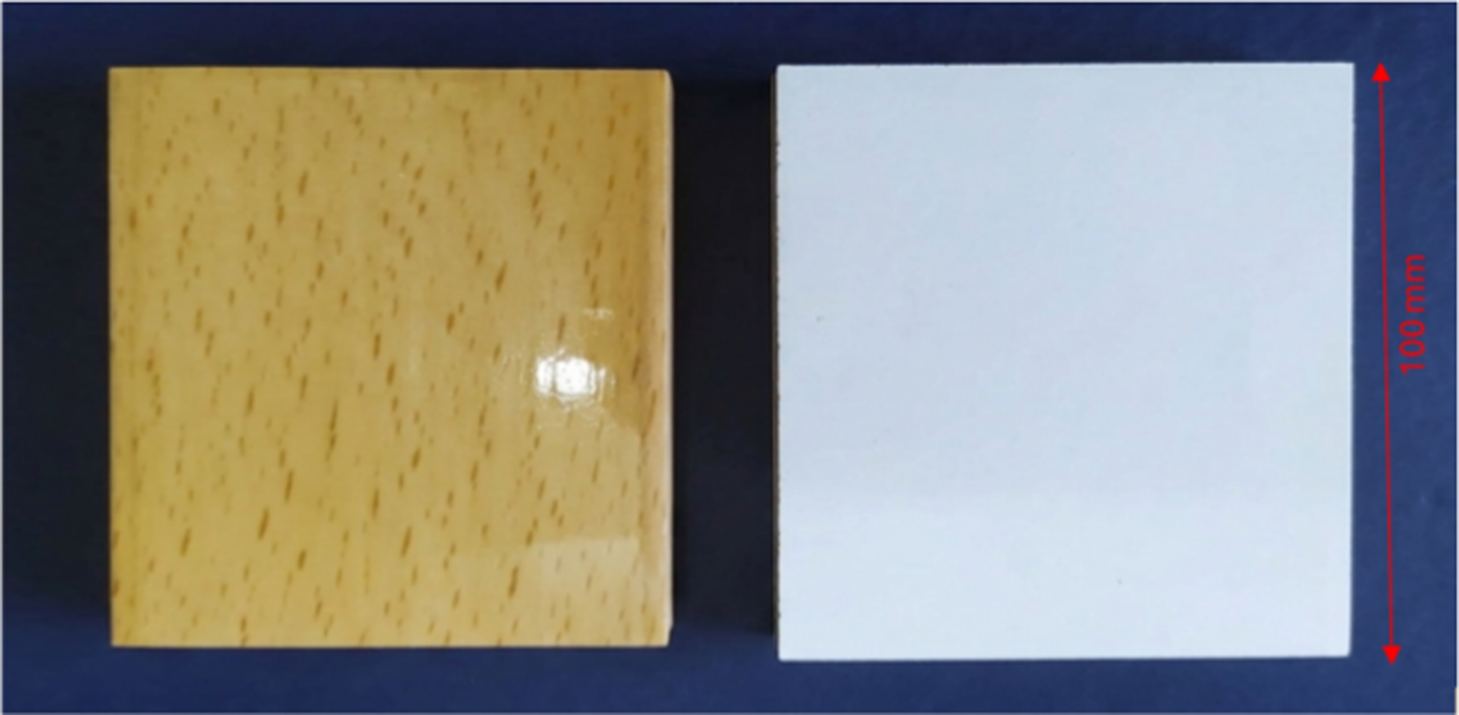
Application of water-based transparent varnish and white pigmented paint on Eastern beech (*F. orientalis* L.) wood specimens.

After application of the final topcoat, all coated specimens were allowed to cure for 7 days under controlled laboratory conditions at 23 ± 2 °C and 50 ± 5% relative humidity. These curing conditions were selected in accordance with ASTM D3924-16 (2019) and were maintained to ensure complete solvent evaporation, film coalescence, and curing of the coating systems prior to mechanical testing. The seven-day conditioning period was considered sufficient to achieve stable film formation and minimize the potential influence of incomplete curing on hardness and scratch resistance measurements.

### Environmental Conditioning Prior to Testing

To guarantee the reproducibility of the mechanical tests, the varnished and painted samples underwent a 7 day conditioning phase at 23 ± 2 °C and 50 ± 5% relative humidity, adhering to the ASTM D3924-16 (2019) standard.^28^ Environmental variations were minimized by performing all subsequent evaluations in a climate-controlled laboratory setting. The finished specimens were then systematically subjected to scratch resistance profiling in conjunction with surface hardness testing.

For each coating formulation, mechanical testing was performed using ten replicate specimens. Measurements were obtained from randomly selected, nonoverlapping locations on each specimen surface to minimize local variability and ensure statistical representativeness. Accordingly, a total of 80 coated specimens were produced for the experimental program (4 varnish systems +4 paint systems; *n* = 10 per coating type). In addition, a total of 160 tests were conducted for the pendulum hardness and scratch resistance. In cases where the data were inconsistent or unsuccessful, new test samples were produced and tested again, in accordance with the standards.

### Scratch Resistance Test

To characterize the mechanical surface performance of the coating film layers, scratch resistance measurements were performed in conjunction with König pendulum hardness testing. The scratch resistance test was used as a complementary method to evaluate coating durability and resistance to localized mechanical damage. To maintain comparability, all measurements were conducted simultaneously on the same specimen surfaces under controlled laboratory conditions.

Scratch resistance of the varnished and painted surfaces was evaluated using a Taber 511 Scratch Tester (Taber Industries, Tonawanda, NY, USA) in accordance with ASTM D7027-20 (2020).^20^ To ensure baseline equilibrium and data reproducibility, all coated substrates were preconditioned at 23 ± 2 °C and 50 ± 5% relative humidity for 24 h before characterization. The scratch test was performed using a hemispherical diamond stylus supplied with the Taber 511 Scratch Tester. The stylus was traversed across the coated surface at a constant speed of 10 mm s^–1^ in accordance with the manufacturer’s operating procedure and ASTM D7027-20 recommendations. The scratch length was maintained constantly for all specimens, and the load was progressively adjusted to determine the critical load corresponding to the onset of visible coating failure. All measurements were performed under identical testing conditions to ensure comparability among coating systems.

Scratch resistance evaluation was performed by drawing a diamond-tipped stylus across the coated surface under a progressively decreasing load profile. The scratch test was performed by using a hemispherical diamond stylus with a tip radius of 1.0 mm. The applied normal force was systematically reduced in a stepwise manner to identify the onset of surface damage: from 5 to 2 N in 0.5 N increments, from 2 to 1 N in 0.25 N increments, and below 1 N in 0.1 N increments until intermittent scratching was discernible. The critical load, defined as the maximum force sustained by the film without causing visible macrodamage or coating failure, was recorded to quantify the scratch resistance of each specimen (Table 1).

**Table 1:** Classification of Scratch Resistance

classification of coating film layers	resistance (N)
1	4.1–5.0
2	2.1–4.0
3	1.6–2.0
4	1.1–1.5
5	0.5–1.0
6	less than 0.5

The physical evaluation of scratch resistance in this study is directly governed by the penetration-depth mechanism. From a mechanistic perspective, as the normal load is progressively applied or altered, it establishes a direct continuum with the instantaneous penetration depth of the diamond stylus into the coating matrix. Under high normal loads (e.g., 5 N), the vertical stress exceeds the cohesive strength of the polymer chains, forcing the stylus to penetrate deeply past the elastic zone. At this maximum penetration depth, the lateral shear stress induces severe plastic deformation, leading to the microtearing and catastrophic rupture of the coating film layer.

Conversely, as the normal load is systematically reduced during the test sequence, the vertical penetration depth decreases. The transition point where the continuous tearing ceases and transforms into intermittent scratching represents the exact threshold where the penetration depth reaches the critical penetration depth. At this specific depth, the viscoelastic recovery of the coating matrix begins to successfully counteract the diamond tip’s geometry, meaning the applied load is no longer sufficient to maintain continuous propagation of film failure. Therefore, the critical load recorded at these intermittent scratch marks serves as the definitive indicator of scratch resistance, precisely defining the maximum depth the polymer matrix can withstand before transitioning from nondestructive elastic compliance to permanent mechanical tearing.

### Scanning Electron Microscopy Analysis

The scratch-induced damage morphology of the coating systems was investigated by using scanning electron microscopy (SEM). Following scratch resistance testing, representative specimens exhibiting characteristic scratch tracks were selected from each coating formulation for a microscopic examination. Small sections containing the scratch groove were carefully cut from the coated panels and conditioned at a laboratory temperature prior to analysis.

Because polymer coatings and wood substrates are electrically nonconductive materials, the specimens were sputter-coated with a thin gold layer to minimize surface charging during SEM observation. SEM imaging was performed using a JEOL JSM-series scanning electron microscope (JEOL Ltd., Tokyo, Japan) operating under high-vacuum conditions at an accelerating voltage of 10–15 kV.

Micrographs were acquired at various magnification levels to evaluate scratch groove morphology, localized plastic deformation, lateral pile-up formation, microcrack initiation, coating fracture behavior, delamination phenomena, and coating–substrate interaction mechanisms. The SEM observations were used as qualitative evidence to support the interpretation of scratch resistance performance and the deformation mechanisms associated with different coating chemistries and coating architectures.

### Pendulum (König) Hardness Test

The surface hardness of the varnished and painted samples was determined via the König pendulum damping test in accordance with ASTM D4366-16 (2021), employing an Erichsen Pendulum Damping Tester (Model 299/300, Germany).^19^ To ensure baseline equilibrium and data reproducibility, all coated substrates were preconditioned at 23 ± 2 °C and 50 ± 5% relative humidity for 24 h before characterization. The measurement involved placing a pendulum, fitted with two hardened steel balls (5 mm diameter, HRC 63), directly onto the coated layer. The instrument automatically logged the total number of oscillations required for the deflection amplitude to decrease from 6° to 3°. Here, an elevated oscillation count corresponds to high surface hardness and enhanced resistance to localized deformation.

### Coating Thickness Measurement

Coating thickness was determined using a destructive cross-sectional method in accordance with ASTM D4138-07a (2022), Procedure A. A V-groove was carefully introduced at predefined measurement locations to facilitate exposure of the coating–substrate interface.^29^ Cross-sectional regions were subsequently analyzed using a Zeiss Axio Scope A1 stereomicroscope (Carl Zeiss Microscopy GmbH, Germany) equipped with ZEN imaging software. Prior to measurement, the optical system was calibrated with a certified stage micrometer to ensure dimensional accuracy (Figure 2).

**Figure 2:**
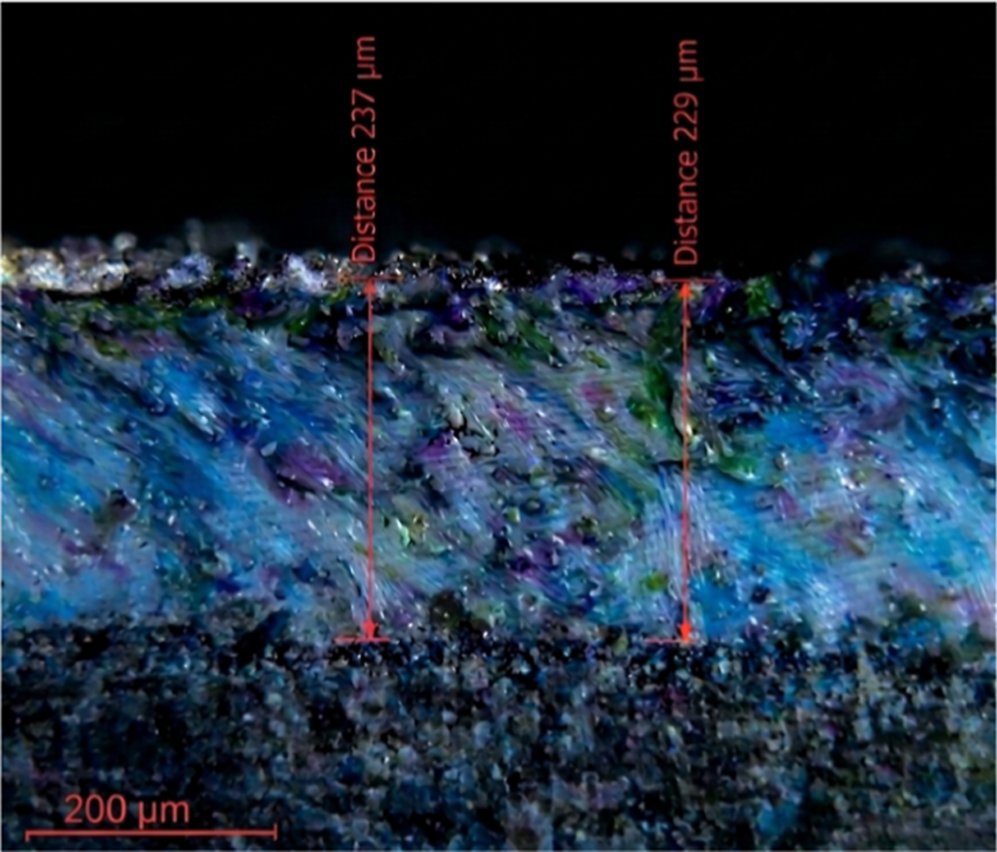
Measurement of white pigmented paint layer thickness using a stereo microscope.

For each coating system, multiple randomly selected cross sections were prepared and evaluated. Film thickness was quantified as the perpendicular distance between the external coating surface and the coating–wood interface. Ten independent measurements (*n* = 10) were performed for each varnish and paint formulation, and the arithmetic mean values were calculated for further evaluation. Measurements were obtained from ten independent coated specimens, with one cross-sectional thickness determination performed on each specimen.

### Schematic Interpretation of Scratch-Induced Damage

A schematic illustration was prepared by using AutoCAD software to visually summarize the proposed damage evolution mechanism occurring during scratch resistance testing. The figure was developed exclusively as a conceptual graphical representation based on the experimental observations obtained from scratch-resistance measurements and scanning electron microscopy (SEM) analyses.

It is intended solely to assist in the interpretation of the penetration process, localized deformation, crack initiation, and coating failure mechanisms observed in the transparent varnish and pigmented-coating systems investigated.

### Experimental Workflow

The workflow illustrates the sequential stages employed in the experimental program (Figure 3). Initially, defect-free eastern beech (*F. orientalis* L.) wood specimens were prepared, sanded, and conditioned under controlled environmental conditions. Subsequently, transparent varnish and pigmented paint systems based on acrylic, polyurethane, water-based, and cellulosic chemistries were applied using a standardized HVLP spraying process.

**Figure 3:**
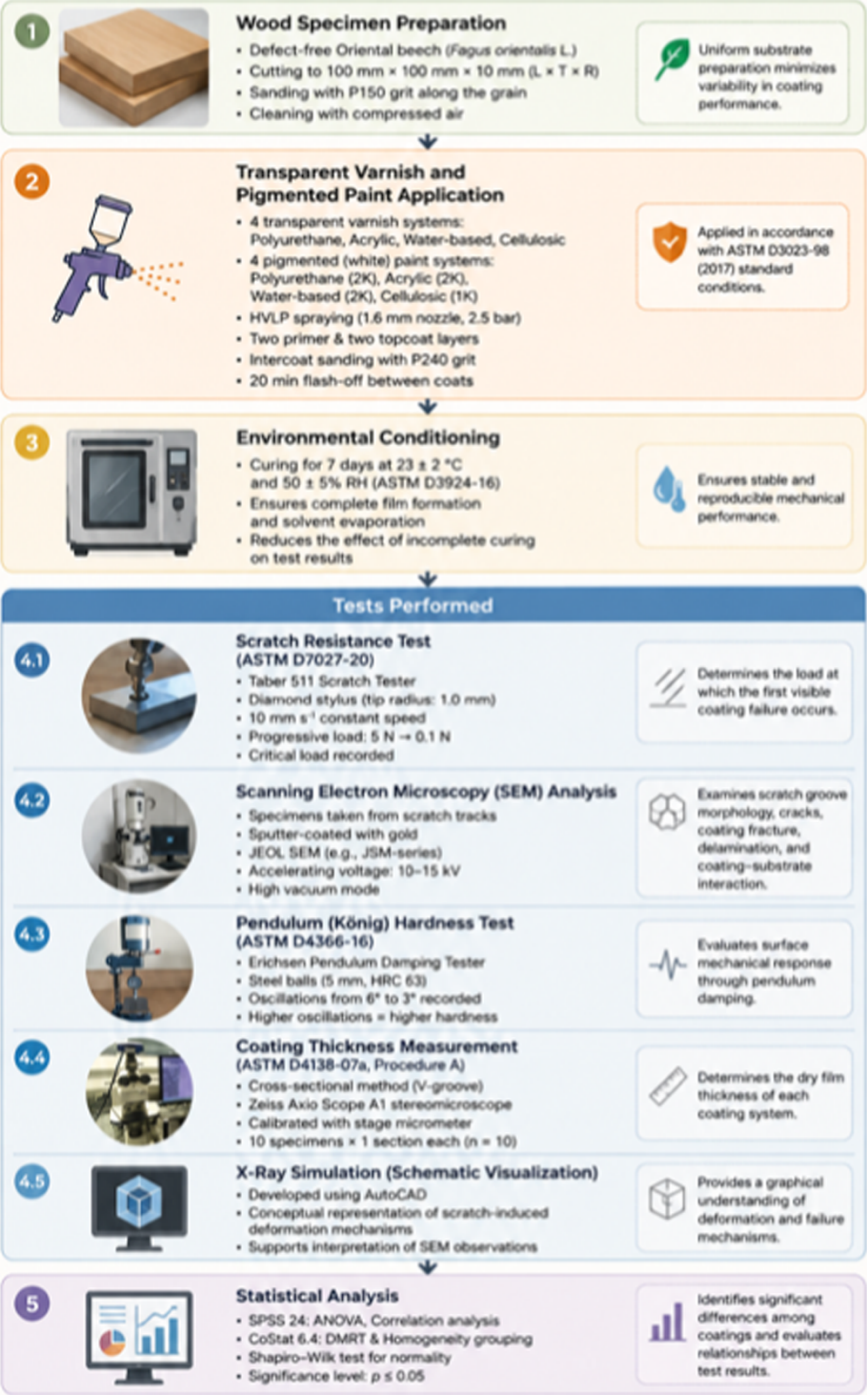
Experimental workflow for evaluating the mechanical surface performance of transparent varnish and pigmented paint coating systems applied to Eastern beech (*F. orientalis* L.) wood panels.

Following coating application, all specimens were conditioned to ensure complete film formation and curing prior to mechanical characterization. The coated surfaces were then evaluated using scratch resistance testing and König pendulum hardness measurements. To further investigate the mechanisms governing coating performance, scratch-induced damage zones were examined by scanning electron microscopy (SEM), while coating thicknesses were determined using a cross-sectional optical microscopy method. In addition, a schematic interpretation simulation was developed to facilitate interpretation of deformation and failure mechanisms observed during scratch loading.

The experimental data obtained from all characterization techniques were subsequently analyzed using statistical methods, including analysis of variance (ANOVA), Duncan’s Multiple Range Test (DMRT), homogeneity grouping, and correlation analysis. This integrated workflow enabled a comprehensive assessment of the influence of coating chemistry and coating architecture on the mechanical surface performance of transparent varnish and pigmented paint systems.

### Statistical Analysis

Statistical analyses were performed using SPSS 24 (IBM Corp., Armonk, NY, USA) and CoStat 6.4 (CoHort Software, Monterey, CA, USA). In SPSS, numerical data and ranking values for each group were obtained; however, the software does not generate homogeneity groupings (e.g., a, ab, bc, cd) in post hoc multiple comparison tests. Therefore, CoStat was employed to identify homogeneous subsets and visualize the statistically equivalent groups. The combined use of both programs enabled a more precise statistical interpretation and clearer differentiation among varnish types in terms of hardness and scratch resistance.

Normality of the data was assessed using the Shapiro–Wilk test.^30^ Analysis of variance (ANOVA) was conducted to evaluate the effects of measurement method and varnish type. Group comparisons were performed using Duncan’s Multiple Range Test (DMRT) and the least significant difference (LSD) method. Pearson correlation analysis was applied to examine the relationship between hardness values and scratch resistance measurements.

The experimental design consisted of eight coating formulations (four transparent varnishes and four pigmented paints), with ten independent replicate specimens prepared for each formulation (*n* = 10). Statistical analyses were performed by using specimen-level mean values obtained from pendulum hardness, scratch resistance, and coating thickness measurements.

## Results

### Measurement Results

The effects of measurement method and coating type on hardness and scratch resistance were evaluated using analysis of variance (ANOVA), and the corresponding statistical results are summarized in Table 2. Analysis of variance (ANOVA) revealed that both the test methods and the coating (varnish and paint) formulation had statistically significant effects on the measured hardness and scratch resistance values (*p* < 0.05). Moreover, significant interaction was observed between the two factors, indicating that the effect of varnish and paint types on hardness and scratch resistance varies depending on the measurement method used.

**Table 2:** ANOVA Results Show the Effects of Testing Methods and Coating Types^a^

factors	sum of squares	degrees of freedom	mean square	*F* value	level of significance
measurement method (A)	272,415.025	1	134,251.102	73,625.682	0.001^a^
coating type (B)	10,967.425	7	1566.775	423.4527	0.001^a^
interaction (AB)	9804.225	7	1400.6036	378.54151	0.001^a^
model	293,186.675	15	19,545.778	5282.6428	0.001^a^
error	532.8	144	3.7		
total	293,719.475	159			

a Statistically significant at the 95% confidence level.

According to the ANOVA results presented in Table 2, all main factors and their interactions significantly affected the hardness measurements (*p* ≤ 0.05). The outcomes of Duncan’s Multiple Range Test (DMRT), conducted to compare differences among measurement methods and varnish types based on the LSD critical value, are summarized in Table 3.

**Table 3:** DMRT Results Show the Effects of Varnish and Paint Types on Hardness and Scratch Resistance Measurement Values^a^

measurement methods	*n*	x̅	LSD
pendulum hardness measurement method	80	85.7	±0.61
scratch resistance measurement method (N)	80	3.2	

transparent varnish types	*n*	x̅	HG	LSD
cellulosic varnished sample	10	33.75	D̂	±1.21
water based varnished sample	10	45.90	C	
polyurethane varnished sample	10	49.65	B	
acrylic varnished sample	10	55.90	A*	

paint types	*n*	x̅	HG	LSD
cellulosic paint sample	10	30.12	D̂	±1.21
water based paint sample	10	42.27	C	
polyurethane paint sample	10	46.02	B	
acrylic paint sample	10	52.27	A*	

a *: the maximum value; ^: the minimum value; HG: homogeneity group; x̅ : mean value.

The DMRT results presented in Table 3 demonstrated statistically significant differences among the coating formulations with respect to their combined hardness and scratch-resistance performance. Across both transparent varnish and pigmented paint systems, a consistent performance hierarchy was observed, following the order: acrylic > polyurethane > water-based > cellulosic. This ranking suggests that the mechanical durability of the coating systems was primarily governed by the chemical structure of the binder matrix and the degree of network formation developed during the curing.

Among the transparent varnish formulations, the acrylic varnish exhibited the highest overall performance index (55.90), followed by polyurethane (49.65), water-based (45.90), and cellulosic varnishes (33.75). A similar trend was observed for the pigmented coating systems, where acrylic paint produced the highest performance value (52.27), whereas cellulosic paint yielded the lowest value (30.12). The statistical consistency observed between varnish and paint groups indicates that resin chemistry exerted a stronger influence on mechanical performance than the coating category alone.

The high behavior of acrylic and polyurethane systems can be attributed to their highly cross-linked polymer architectures, which enhances cohesive strength, load distribution capability, and resistance to localized deformation. Previous investigations have demonstrated that increased cross-link density improves hardness, scratch resistance, abrasion resistance, and overall mechanical stability in wood coating systems. Acrylic and polyurethane coatings are known to develop dense three-dimensional polymer networks that effectively restrict molecular mobility under mechanical loading, thereby increasing resistance to surface damage and crack propagation. In contrast, water-based coatings generally rely on particle coalescence mechanisms and often exhibit lower effective cross-link densities, whereas cellulosic coatings predominantly display thermoplastic characteristics associated with reduced cohesive strength and lower resistance to permanent deformation. These observations are in good agreement with previous studies reported for wood coatings and polymeric protective films.^16,31–34^

An additional outcome of particular importance is the systematic performance advantage observed for transparent varnish systems compared with their pigmented paint counterparts within the same resin chemistry. For all coating chemistries investigated, transparent varnishes exhibited higher performance values than the corresponding pigmented coatings. This behavior suggests that pigment incorporation modifies the internal microstructure of the coating film by introducing additional particle–matrix interfaces that may act as localized stress concentration regions during mechanical loading. Similar observations have been reported in coating science, where heterogeneous pigment dispersion may promote microcrack initiation, localized plastic deformation, and energy dissipation, ultimately reducing resistance to scratch-induced failure and surface damage.^10,35,36^

### DMRT Comparative Results of Pendulum Hardness and Scratch Resistance Measurements

Table 4 summarizes the results of Duncan’s Multiple Range Test (DMRT) conducted to evaluate differences in mechanical surface performance among the transparent varnish and pigmented paint systems investigated. Pendulum hardness and scratch resistance measurements were used to assess the resistance of the coating films to surface deformation and scratch-induced damage. The obtained data were subsequently classified according to coating category and resin chemistry to identify statistically significant differences among the investigated formulations.

**Table 4:** DMRT Results for Pendulum Hardness and Scratch Resistance Measurements of Transparent Varnish and Pigmented Paint Coating Systems^a^

	measurement methods					
	pendulum hardness measurement method			scratch resistance measurement method (N)		
transparent varnish types	95% CI	mean ± SD	HG	95% CI	mean ± SD	HG
cellulosic varnished sample	61.91–67.49	64.70 ± 2.79	D̂	2.67–2.93	2.80 ± 0.13	D̂
water based varnished sample	88.28–91.32	88.80 ± 2.52	C	3.10–2.90	3.00 ± 0.10	C
polyurethane varnished sample	93.39–98.01	95.70 ± 2.31	B	3.48–3.72	3.60 ± 0.12	B
acrylic varnished sample	104.72–110.88	107.80 ± 3.08	A*	3.85–4.15	4.00 ± 0.15	A*

pigmented paint types	95% CI	mean ± SD	HG	95% CI	mean ± SD	HG
cellulosic paint sample	55.09–60.31	57.70 ± 2.61	D̂	2.40–2.70	2.55 ± 0.15	D̂
water based paint sample	79.31–84.29	81.80 ± 2.49	C	2.65–2.85	2.75 ± 0.10	C
polyurethane paint sample	86.35–91.05	88.70 ± 2.35	B	3.23–3.47	3.35 ± 0.12	B
acrylic paint sample	98.50–103.10	100.80 ± 2.30	A*	3.62–3.88	3.75 ± 0.13	A*
LSD ± 1.55						

a Note: *: the maximum value; ^: the minimum value; HG: homogeneity group; CI: confidence interval; SD: standard deviation; values are presented as mean ± standard deviation (*n* = 10). Homogeneity groups were determined using Duncan’s Multiple Range Test at α = 0.05.

The DMRT results presented in Table 4 revealed statistically significant differences among the coating systems for both pendulum hardness and scratch resistance performance (in addition to DMRT groupings, 95% confidence intervals were calculated for all measured variables and used to verify the robustness of statistical comparisons, *p* ≤ 0.05). A consistent ranking was observed for both transparent varnish and pigmented paint formulations, following the order: acrylic > polyurethane > water-based > cellulosic. This trend indicates that the mechanical surface performance of the coatings was strongly influenced by the chemical nature of the binder system and the resulting polymer network structure.

Among the transparent varnish systems, the acrylic coating exhibited the highest pendulum hardness (107.80) and scratch resistance (4.00 N), whereas the cellulosic varnish produced the lowest values for both properties. A similar performance hierarchy was observed for the pigmented coating systems, where acrylic paint showed high hardness (100.80) and scratch resistance (3.75 N), while the cellulosic paint exhibited the weakest mechanical performance.

The high performance of the acrylic and polyurethane systems can be attributed to their highly developed polymer network structures and enhanced film-forming characteristics. Previous studies have demonstrated that the mechanical performance of wood coating systems is strongly governed by cross-link density, intermolecular interactions, and coating cohesion, all of which directly influence hardness, scratch resistance, and deformation behavior.^36–40^ Acrylic coatings generally form dense and mechanically stable films with strong intermolecular interactions, providing improved resistance against surface deformation and scratch-induced damage. Similarly, polyurethane systems benefit from extensive three-dimensional network formation through urethane linkages, resulting in increased load-bearing capacity and resistance to mechanical failure. Comparable observations have been reported in polyurethane-acrylate coating systems, where highly cross-linked polymer architectures significantly improved scratch and abrasion resistance.^37,41^ In contrast, water-based coatings typically depend on particle coalescence mechanisms during film formation, which may generate lower cross-link densities than solvent-borne thermosetting systems, thereby reducing their resistance to localized mechanical stresses.^39^ Cellulosic coatings, which primarily exhibit thermoplastic behavior, generally possess lower cohesive strength and reduced resistance to deformation, making them more susceptible to surface damage under scratch loading conditions.^36^

An additional finding of particular importance is the consistent performance advantage of transparent varnish systems over their pigmented paint counterparts within the same resin chemistry. Similar trends have been reported in coating science, where the incorporation of pigment particles and fillers modifies the internal stress distribution of the coating matrix and can significantly influence mechanical durability.^37–41^ For all four coating chemistries investigated in the present study, transparent varnishes exhibited higher pendulum hardness and greater scratch resistance than pigmented coatings. This behavior indicates that coating architecture plays a critical role in determining surface durability beyond resin chemistry alone. The introduction of pigment particles creates additional interfacial regions within the coating film, which may act as localized stress concentration sites during mechanical loading. These heterogeneities can promote microcrack initiation, localized plastic deformation, and energy dissipation processes, thereby accelerating coating damage evolution and reducing resistance to scratching. Similar scratch-induced fracture and deformation mechanisms associated with heterogeneous coating structures have been documented in polymer-based coating systems subjected to mechanical loading.^37,38^

The strong agreement between the pendulum hardness and scratch resistance rankings further suggests that both methods consistently reflected the intrinsic mechanical integrity of the coating films (Figure 4). Previous studies have shown that coatings exhibiting higher cohesive strength, stronger polymer network formation, and improved resistance to surface deformation generally demonstrate high performance in both hardness and scratch-resistance evaluations.^36,37,40,41^ The present findings therefore indicate that the coating chemistry and coating architecture jointly govern the mechanical surface performance of wood finishing systems. Among the investigated formulations, acrylic and polyurethane transparent varnishes provided the most favorable combination of hardness and scratch resistance, suggesting that highly cross-linked and structurally homogeneous coating systems offer improved protection against scratch-induced surface degradation and long-term mechanical wear. These interpretations are primarily supported by the SEM observations presented in Figure 7. In addition, the schematic illustration shown in Figure 6 serves as a conceptual aid for visualizing the coating deformation and damage mechanisms associated with the scratch loading.

**Figure 4:**
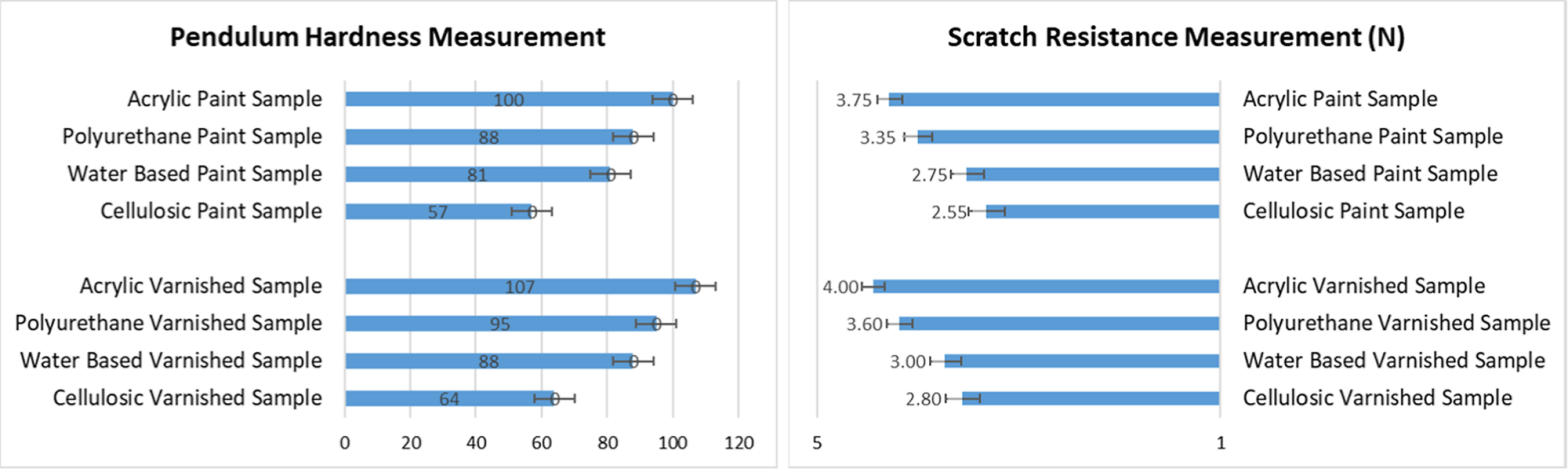
Comparison of data obtained from scratch resistance tests and pendulum hardness tests of pigmented paints and transparent varnishes.

## Discussion

### Coating Thickness Characteristics of Transparent Varnish and Pigmented Paint Systems

The thickness of the varnish layer applied to Eastern beech (*F. orientalis* L.) samples was measured using a Zeiss Axio Scope A1 stereo microscope (Figure 2). High-resolution images captured via the microscope’s integrated camera system were analyzed using ZEN imaging software, enabling precise measurement of the varnish film thickness at the micron (μm) scale. The results of these measurements are presented in Table 5.

**Table 5:** Micron-Scale Thickness of Pigmented Paint and Transparent Varnish Layers on Eastern Beech (*F. orientalis* L.) (μm)

coating type	cellulosic (μm)	water based (μm)	polyurethane (μm)	acrylic (μm)
transparent varnish layer thickness ( x̅ )	231.6	237.3	229.7	222.4
pigmented paint layer thickness ( x̅ )	238.5	231.3	223.1	229.1

Microscopic measurements presented in Table 6 showed that the average dry film thickness of the investigated coating systems ranged from approximately 222 to 238 μm. Although minor variations in coating thickness were observed among the transparent varnish and pigmented paint formulations, all coatings were applied under identical ASTM-standardized processing conditions using the same spray parameters, application sequence, and curing protocol. Consequently, the measured thickness values remained within a relatively narrow range suitable for comparative evaluation of mechanical surface performance.

**Table 6:** Correlation Analysis between Pendulum Hardness and Scratch Resistance Measurements

groups included in the analysis	the Pearson correlation coefficient (*r*)	number of samples (*n*)	the *P*-value
the pendulum hardness measurement method and the scratch resistance measurement method	0.99	160	0.001^a^

a Statistically significant at *p* < 0.01.

The maximum thickness difference observed among the coating systems was approximately 16 μm, corresponding to less than 8% of the average film thickness. Such variations are considerably smaller than the overall coating thickness and are unlikely to represent the primary factor governing the observed differences in pendulum hardness and scratch resistance. Previous studies have demonstrated that, when coating thickness remains within a comparable range, the mechanical behavior of wood coating systems is predominantly controlled by polymer chemistry, cross-link density, film cohesion, and coating microstructure rather than small fluctuations in dry film thickness.^16,18,42–45^

### Pendulum (König) Hardness Results

Visual examination of the coating surfaces following König pendulum hardness testing revealed no observable coating fracture, crack formation, delamination, or permanent surface damage. Unlike scratch resistance testing, which intentionally induces localized mechanical failure to evaluate coating durability, the pendulum hardness method evaluates surface mechanical response through oscillatory damping behavior without generating visible coating disruption. These observations support the suitability of the pendulum method for repeated hardness evaluation of coated wood surfaces while preserving coating integrity under the testing conditions employed in the present study.^18^

SEM examination of the coating surfaces after König pendulum hardness testing revealed no detectable coating fracture, crack initiation, delamination, or permanent surface deformation (Figure 5). Unlike scratch resistance testing, which intentionally generates localized damage to evaluate coating durability, the pendulum method assesses surface mechanical response through oscillatory damping without producing observable coating failure. These observations indicate that the test did not adversely affect coating integrity under the experimental conditions employed in the present study.

**Figure 5:**
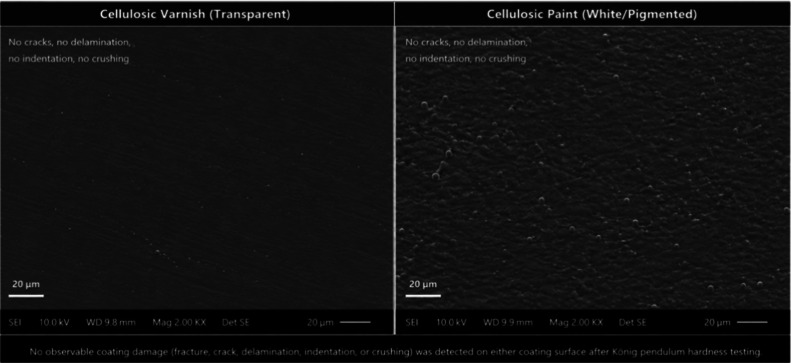
SEM observations of cellulosic transparent varnish and cellulosic pigmented coating surfaces following König pendulum hardness testing.

Figure 5 reveals a clear difference in surface morphology between the transparent and pigmented cellulosic coating systems despite the absence of visible mechanical damage after König pendulum testing. The transparent cellulosic varnish exhibited a relatively smooth and homogeneous surface, reflecting the formation of a continuous polymer film with limited microstructural heterogeneity. In contrast, the pigmented cellulosic coating displayed a noticeably rougher surface characterized by localized protrusions and microscale topographical variations. This behavior can be attributed to the presence of pigment and extender particles dispersed within the coating matrix. During solvent evaporation and film formation, pigment particles may locally modify film leveling behavior and generate surface irregularities associated with particle packing and pigment–binder interactions. Similar differences between transparent and pigmented coating systems have been reported in previous studies, where pigment incorporation increased surface roughness and microstructural heterogeneity without necessarily inducing coating defects like cracking or delamination.^31–39^

### Scratch Resistance Results and Schematic Simulation

Scratch resistance measurements revealed clear differences among the investigated coating systems, indicating that both the resin chemistry and coating architecture strongly influenced the resistance of coating films to localized mechanical damage. Consistent with the pendulum hardness results, the scratch resistance ranking followed the order acrylic > polyurethane > water-based > cellulosic for both transparent varnish and pigmented paint systems. This agreement suggests that coatings possessing greater resistance to surface deformation also exhibit an improved resistance against stylus-induced fracture and material removal.

The high scratch resistance exhibited by acrylic and polyurethane coatings can be primarily attributed to their highly cross-linked polymer network structures. In thermosetting acrylic systems, extensive intermolecular interactions and dense polymer chain packing generate coatings with an elevated cohesive strength and enhanced resistance to plastic deformation. Similarly, polyurethane coatings derive their mechanical performance from urethane linkages and three-dimensional cross-linked architectures that effectively distribute externally applied stresses throughout the coating matrix. Consequently, highly cross-linked coating systems generally exhibit improved resistance to crack initiation, groove formation, and coating fracture during scratch testing.

In contrast, water-based coatings exhibited intermediate scratch-resistance values. Although modern waterborne formulations can develop a satisfactory mechanical performance after film formation, their microstructure is largely governed by particle coalescence mechanisms. The resulting polymer networks frequently possess lower cross-link densities and greater free-volume content than solvent-borne thermosetting systems. These structural characteristics may facilitate localized viscoelastic deformation under concentrated loads, thereby reducing resistance to stylus penetration and scratch propagation. Similar relationships between reduced cross-link density and lower scratch resistance have been reported for waterborne wood coating systems in previous investigations.^33,34,46,47^

The lowest scratch resistance values were consistently observed in the cellulosic coatings. Cellulose-based finishing systems primarily exhibit thermoplastic behavior and generally lack the highly interconnected network structures characteristic of acrylic and polyurethane coatings. As a result, the coating matrix possesses a lower cohesive strength and reduced resistance to stress concentration. Under scratch loading, these coatings are more susceptible to localized plastic flow, crack propagation, and material displacement, leading to an earlier onset of coating damage. Comparable observations have been reported in the wood finishing literature, where cellulosic coatings consistently demonstrated inferior resistance to abrasion, indentation, and scratch-induced surface degradation compared with thermosetting coating systems.^48,49^

An additional finding of particular significance was the systematic performance advantage of transparent varnishes over pigmented paint systems within the same resin chemistry. For all investigated coating chemistries, transparent varnishes exhibited higher critical scratch loads than their corresponding pigmented coatings. This behavior indicates that coating architecture contributes substantially to scratch resistance beyond the influence of binder chemistry alone. The incorporation of pigment particles introduces numerous particle–matrix interfaces throughout the coating structure. These heterogeneous regions may act as localized stress concentration sites during scratch loading, facilitating microcrack initiation and localized fracture processes. Previous coating science studies have shown that pigment volume concentration, particle dispersion quality, and pigment–binder interfacial adhesion significantly influence scratch resistance and damage evolution behavior.^10,16,22,31,50^

The schematic simulations shown in Figure 6 were created using AutoCAD software as conceptual graphical representations of the scratch-induced damage mechanisms inferred from scratch resistance testing and SEM surface observations. In transparent polyurethane varnish systems, scratch loading is predominantly accommodated through localized coating displacement and limited microcrack formation, indicating a relatively homogeneous stress distribution within the coating film. In contrast, pigmented polyurethane coatings exhibit more pronounced fracture features, including crack development, coating fragmentation, and intensified coating–substrate interaction. The presence of pigment particles and additional internal interfaces likely promotes heterogeneous stress transfer during stylus penetration, accelerating the initiation of coating failure. These observations are consistent with established scratch damage models reported for polymer coating systems, where crack initiation, lateral pile-up formation, coating fracture, and interfacial damage progressively develop as applied stress exceeds the cohesive strength of the coating matrix.

**Figure 6:**
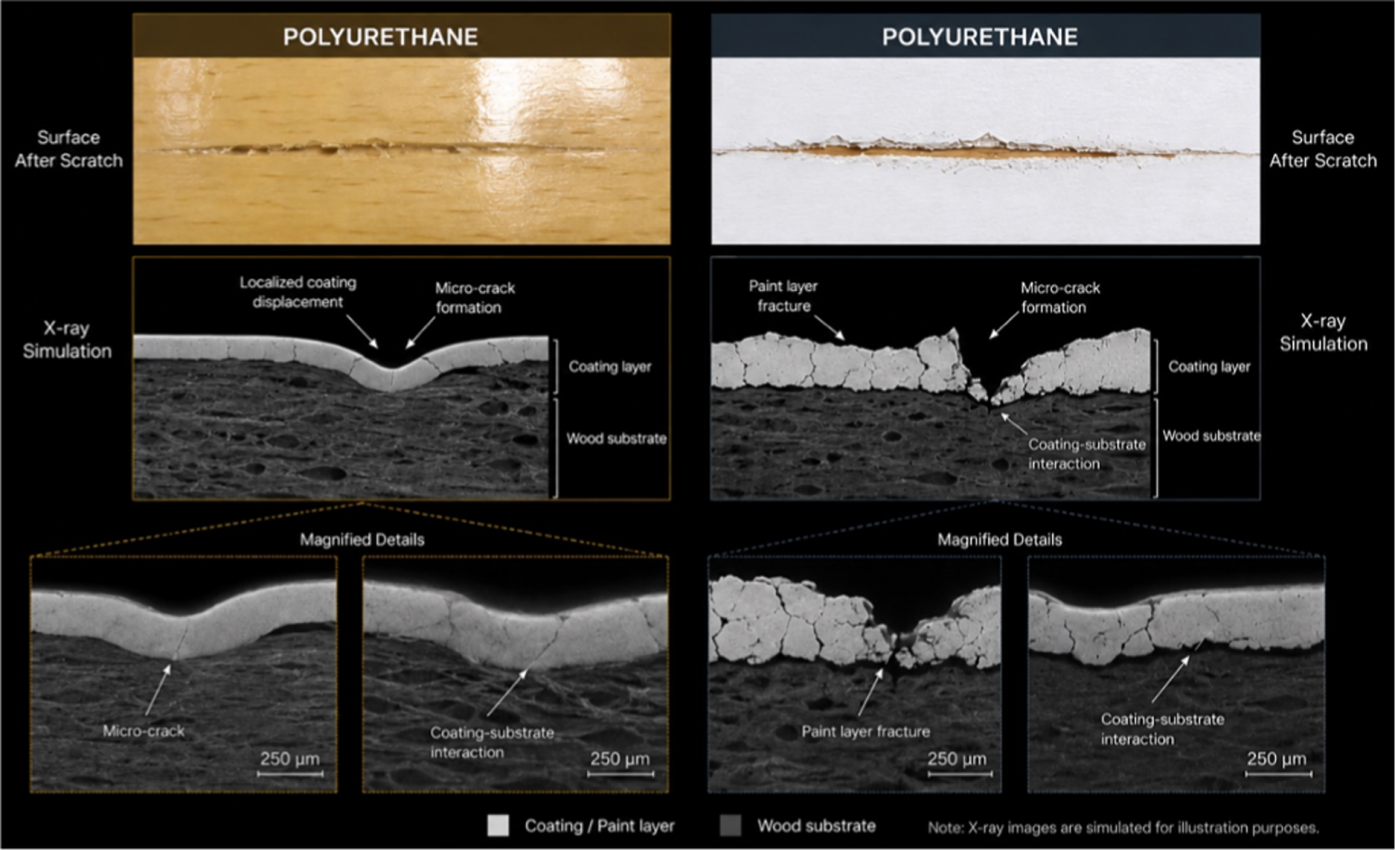
Conceptual schematic illustration of the scratch-induced damage evolution mechanism in transparent varnish and pigmented coating systems.

### SEM Analysis of Scratch-Induced Damage Mechanisms

The SEM micrographs presented in Figure 7 provide valuable insights into the damage evolution mechanisms occurring within transparent varnish and pigmented paint systems during scratch resistance testing. Distinct differences in scratch groove morphology were observed between the two coating architectures, indicating that the mechanical response of the surface is strongly influenced by both the coating composition and structural configuration.

**Figure 7:**
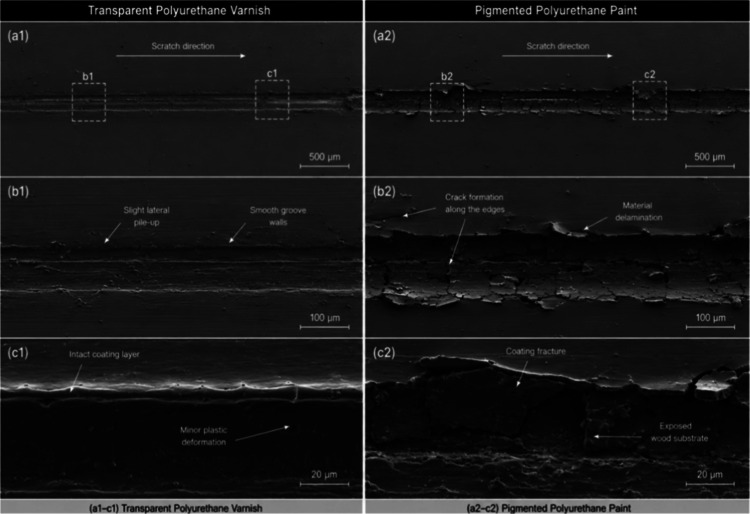
SEM micrographs of scratch tracks formed on transparent polyurethane varnish and pigmented polyurethane paint coatings after scratch resistance testing according to ASTM D7027-20.

The transparent polyurethane varnish exhibited a relatively smooth and continuous scratch track characterized by limited plastic deformation and minimal disruption of the surrounding coating material (Figure 7a1,b1,c1). Only minor lateral pile-up formation was observed along the groove edges, suggesting that the applied load was accommodated primarily through elastic–plastic deformation of the coating film rather than through extensive fracture processes. The absence of significant crack propagation and coating detachment indicates strong internal cohesion within the varnish layer as well as effective adhesion at the coating–substrate interface.^31^

In contrast, the pigmented polyurethane paint system displayed a more complex damage morphology. The scratch track was accompanied by pronounced lateral material displacement, localized coating fracture, and the initiation of microcracks extending from the groove boundaries (Figure 7a2,b2,c2). These features indicate that the applied mechanical stress exceeded the local strain tolerance of the coating, resulting in stress concentration and crack development within the pigmented film structure. Furthermore, partial delamination zones were detected adjacent to the scratch path, revealing localized failure of the coating–substrate interface under loading conditions.

The observed differences in deformation behavior can be attributed to variations in the coating architecture. The presence of pigment particles and additional interfaces within the paint system may act as potential stress concentration sites, facilitating the crack initiation and propagation during scratching. Conversely, the more homogeneous structure of the transparent varnish appears to promote a more uniform stress distribution, thereby reducing the susceptibility to fracture and delamination.

Overall, the SEM observations are consistent with the scratch resistance results and provide direct evidence that the coating architecture significantly influences scratch-induced damage evolution. The high resistance of the transparent polyurethane varnish was associated with reduced groove deformation, limited crack formation, and improved coating integrity, whereas the pigmented polyurethane coating exhibited more pronounced fractures, delamination, and localized damage mechanisms under identical testing conditions.

### Comparison of Pendulum Hardness and Scratch Resistance Measurements

To further evaluate the consistency of the mechanical performance assessments obtained from the two testing techniques, Pearson’s correlation analysis was performed between pendulum hardness and scratch resistance measurements. The results are summarized in Table 6.

The analysis revealed an exceptionally strong positive correlation between pendulum hardness and scratch resistance values (*r* = 0.99, *p* < 0.01). This result indicates that coatings exhibiting higher resistance to pendulum damping also demonstrated a greater resistance to scratch-induced surface damage. The strong statistical agreement suggests that both methods consistently reflect the intrinsic mechanical integrity of the investigated coating systems.

From a materials science perspective, the observed relationship can be attributed to the common dependence of both properties on the internal structure of the polymer-coating network. Coatings possessing higher cross-link density, stronger intermolecular interactions, and greater cohesive strength are expected to exhibit increased resistance to both oscillatory surface deformation and localized scratch penetration. Consequently, the ranking obtained from pendulum hardness measurements closely paralleled that derived from scratch resistance testing across all transparent varnish and pigmented paint formulations.

Coating systems exhibiting higher pendulum hardness values generally displayed reduced crack formation, lower levels of coating fracture, and less severe deformation within the scratch-groove region. Conversely, coatings with lower hardness values showed an increased susceptibility to localized damage and crack propagation. These observations demonstrate that the statistical relationship identified through the correlation analysis is supported by the actual damage mechanisms occurring within the coating films.^51^

Although a strong positive correlation was observed between pendulum hardness and scratch resistance, the two methods assess different aspects of coating performance (Figure 8). The König pendulum test primarily characterizes resistance to surface deformation through viscoelastic damping behavior, whereas the scratch resistance test directly evaluates resistance to localized mechanical damage, crack initiation, coating fracture, and material removal. The observed correlation suggests that both properties are governed by similar structural factors, including polymer chemistry, cross-link density, and coating cohesion. Nevertheless, the information provided by each method remains complementary rather than redundant. From an industrial perspective, pendulum hardness testing offers a rapid and nondestructive assessment of coating hardness, while scratch resistance testing provides additional insight into the durability of coating systems under concentrated mechanical loading. Consequently, the combined use of both techniques enables a more comprehensive evaluation of long-term coating performance on wood substrates.

**Figure 8:**
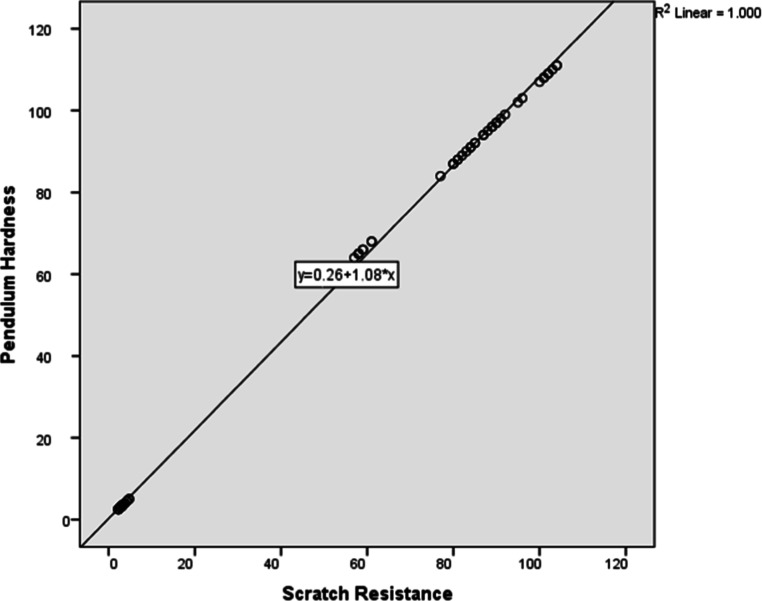
Correlation between pendulum hardness and scratch resistance values of transparent varnish and pigmented paint coatings.

## Conclusions

This study comparatively evaluated the mechanical surface performance of four transparent varnish systems and four pigmented paint systems applied to Eastern beech (*F. orientalis* L.) wood panels using König pendulum hardness testing, scratch resistance measurements, scanning electron microscopy (SEM), and statistical analysis.

The experimental results demonstrated that the coating chemistry significantly affected both hardness and scratch resistance performance. For both transparent varnish and pigmented paint groups, the performance ranking consistently followed the order: acrylic > polyurethane > water-based > cellulosic. The highest pendulum hardness and scratch resistance values were obtained from acrylic coatings, followed by polyurethane systems, whereas cellulosic coatings exhibited the lowest resistance to mechanical loading.

A second major finding was the consistent superiority of transparent varnish systems over pigmented paint systems within the same resin chemistries. Statistical evaluation confirmed that transparent coatings produced higher hardness and scratch resistance values than their pigmented counterparts. This behavior indicates that the coating architecture contributes substantially to surface durability, in addition to binder chemistry. The presence of pigment particles appears to introduce microstructural heterogeneities that promote localized stress concentrations and reduce the resistance to scratch-induced damage.

The strong agreement observed between pendulum hardness and scratch resistance measurements indicates that both methods effectively reflected the intrinsic mechanical integrity of the investigated coating systems. Furthermore, SEM observations revealed clear differences in scratch-induced damage evolution among coating formulations, including variations in crack initiation, localized deformation, coating fracture, and coating–substrate interaction mechanisms. The microscopic findings provided direct evidence that highly cross-linked and structurally homogeneous coatings exhibited greater resistance to mechanical deterioration.

The results further demonstrated that coating performance is governed by the combined effects of polymer chemistry, cross-link density, coating cohesion, and microstructural homogeneity. Acrylic and polyurethane systems benefited from more developed polymer network structures, resulting in improved load-bearing capacity and resistance to surface damage, whereas water-based and particularly cellulosic systems exhibited a lower resistance to localized mechanical stresses.

From a sustainability perspective, the findings suggest that coating selection should be based not only on the formulation type but also on long-term mechanical durability. Coating systems capable of maintaining surface integrity under mechanical loading are expected to reduce the maintenance frequency, refinishing requirements, material consumption, labor demand, and associated environmental burdens throughout the service life of wood products. Consequently, acrylic and polyurethane transparent varnish systems may represent advantageous coating solutions for extending the product lifespan and improving resource efficiency in wood and furniture applications.
